# The Famine and Plague in India

**Published:** 1897-02

**Authors:** 


### THE FAMINE AND PLAGUE IN INDIA.

Dispatches from the East tell of unprecedented suffering and distress occasioned by the double visitation of famine and the plague. While the people in some of the most thickly settled portions of India were in the midst of the former the latter appeared. The *British Medical Journal* says: "The Bombay Government is face to face with the most appalling calamity which has befallen India for the past two centuries, namely, malignant plague and widespread famine. The famine can and will be ameliorated by charity, but no sanitary or medical skill can be bought whereby the disease can be stayed. The death-rate of the city stands at the fearful rate of 200 per 1,000; and there is no indication that the crisis is yet reached; in fact everything points the other way, for instead of the stability of numbers, which is maintained for a week or so, when the maximum of the death-rate is at its climax, we have during the past two weeks a wide diversity.

"The published record of deaths from plague during the past seven weeks is: 49, 51, 53, 67, 64, 173, 259. Nor is this all; for the mortality returns for 'remittent fever' during the week ending December 18, 1896, amounts to the unparalleled total of 363. In other words, 52 persons a day are dying of a fever which at its worst affords but a relatively moderate death-rate. Plague may be said to be killing its hundreds, 'remittent fever' its thousands; a truly ridiculous legend, and one bearing out the inaccuracy of the numbers of the cases of plague in official returns."

And there is no prospect of early relief. Suffering from the famine and the mortality from the bubonic plague or malignant polyadenitis are said to be at present on the increase. The flight of nearly a million people from the plague-stricken district has justly created alarm throughout Western Asia and even in Europe. Sanitary officials of the East, however, are on the alert, and are observing all energetic quarantine measures to prevent the spread of the epidemic.

---

ONE can hardly help being a little surprised in this *fin de siècle* period at the late unpleasantness in medical circles at Augusta over the address delivered by Dr. Jos. Eve Allen at the opening of the Medical College of Georgia upon the "Evolution of Modern Medicine" (published in our November issue). The addres was variously construed by those who heard it. Some said it was an attack upon our sacred Bible and our cherished faith. The faculty of the college in regular meeting resolved that on future similar occasions no reference to religion would be tolerated in any of the speakers; and furthermore that all lectures on all subjects should be strictly in accordance with the teachings of the Bible, and that any professor in any way violating this injunction would thereby forfeit his chair. The matter was finally submitted to the Trustees for final adjustment, to whom Dr. Allen also made a clear statement of his position, and the Trustees officially terminated the difficulty (December 28th) with the resolution that "while according full freedom of thought to every one, they request that professors in discharging their duties in this college avoid all unnecessary reference to questions in dispute between science and religion."

All of which is a veritable tempest in a teapot; which, however, has done very much more harm than would the original address of Dr. Allen if left unchallenged. We have read carefully the address which precipitated this controversy, and we fail to find in it anything which could convict Dr. Allen of being a violent heretic, or of being any less Christian than his accusers. The address was an elaboration of this statement: *The evolution of scientific medicine has been one continuous struggle with dogmatic theology.* It was easy for the speaker to sustain this theme, because he had only to cite certain illustrations from the history of medicine, such as the miracle cure, demoniacal possession, the battle for dissection, public sanitation, the use of anesthetics, etc. The author could easily have multiplied this list. Dr. Allen's statements may be verified if true, or disproven if not true, by any one who will take the trouble to investigate them, but by no sort of interpretation may they be said to assail the impregnable principles of the Christian's faith. Religion, pure and undefiled, is one thing, always the same, yesterday, to-day, and forever; theology and theologians, true and false, are quite another. Religion to-day, as well as in the past, has had occasion to exclaim, "Save me from my friends."

---

THIS number is the last of Volume XIII. We have tried to give our readers a clear, practical and conservative journal, without any blowing of trumpets or fictitious representations as to circulation, or chromo-inducements to subscribe. Nor have we considered it either proper or desirable to introduce literary, scientific, railway, insurance, culinary, metaphysical, sporting and other features. Woman's, children's, and poetical departments have been absolutely interdicted. These and other features above named have no legitimate place in the busy doctor's medical journal. We have some improvements in view for the next volume, and we will endeavor to make it better than the one now ended. Now is the time to subscribe or pay up back dues.

